# FBXO21 mediated degradation of p85α regulates proliferation and survival of acute myeloid leukemia

**DOI:** 10.1038/s41375-023-02020-w

**Published:** 2023-09-09

**Authors:** Kasidy K. Dobish, Karli J. Wittorf, Samantha A. Swenson, Dalton C. Bean, Catherine M. Gavile, Nicholas T. Woods, Gargi Ghosal, R. Katherine Hyde, Shannon M. Buckley

**Affiliations:** 1 Department of Internal Medicine, Division of Hematology & Hematopoietic Malignancies, University of Utah, Salt Lake City, UT USA; 2 Huntsman Cancer Institute, University of Utah, Salt Lake City, UT USA; 3 Department of Genetics, Cell Biology and Anatomy, University of Nebraska Medical Center, Omaha, NE USA; 4 Fred and Pamela Buffett Cancer Center, University of Nebraska Medical Center, Omaha, NE USA; 5 Department of Biochemistry and Molecular Biology, University of Nebraska Medical Center, Omaha, NE USA; 6 Department of Oncological Sciences, University of Utah, Salt Lake City, USA; 7 Eppley Institute, University of Nebraska Medical Center, Omaha, NE USA

**Keywords:** Oncogenes, Cytokines

## Abstract

Acute myeloid leukemia (AML) is a heterogeneous disease characterized by clonal expansion of myeloid blasts in the bone marrow (BM). Despite advances in therapy, the prognosis for AML patients remains poor, and there is a need to identify novel molecular pathways regulating tumor cell survival and proliferation. F-box ubiquitin E3 ligase, FBXO21, has low expression in AML, but expression correlates with survival in AML patients and patients with higher expression have poorer outcomes. Silencing FBXO21 in human-derived AML cell lines and primary patient samples leads to differentiation, inhibition of tumor progression, and sensitization to chemotherapy agents. Additionally, knockdown of FBXO21 leads to up-regulation of cytokine signaling pathways. Through a mass spectrometry-based proteomic analysis of FBXO21 in AML, we identified that FBXO21 ubiquitylates p85α, a regulatory subunit of the phosphoinositide 3-kinase (PI3K) pathway, for degradation resulting in decreased PI3K signaling, dimerization of free p85α and ERK activation. These findings reveal the ubiquitin E3 ligase, FBXO21, plays a critical role in regulating AML pathogenesis, specifically through alterations in PI3K via regulation of p85α protein stability.

## Introduction

Post-translational regulation of hematopoietic differentiation by the ubiquitin proteasome system, specifically the substrate-recognizing ubiquitin E3 ligases is an important direction in unraveling molecular mechanisms regulating cell fate decisions in normal and malignant hematopoiesis [1, 2]. The FBOX family of proteins are a group of approximately 69 ubiquitin E3 ligases that function as the substrate recognition component of the SKP1-CUL1-FBOX (SCF) complex [3, 4]. Of the FBOX family of proteins only 15 of the 69 have known roles in normal and malignant hematopoiesis [5]. Analysis of AML expression datasets revealed one of the FBOX E3 ubiquitin ligases, *FBXO21*, is differentially expressed in leukemia compared to normal bone marrow (BM). Interestingly, low expression of *FBXO21* correlates with better overall survival in AML patients [6]. Little is known about the molecular mechanism of FBXO21, and it has not yet been studied within the context of malignant hematopoiesis.

FBXO21 has two known substrates: EP300-inhibitor of differentiation 1 (EID1) and Apoptosis signal-regulating kinase 1 (ASK1), which were identified in 293 T and RAW264.7 cells, respectively [7–10]. EID1 interacts with RB1 and EP300 leading to maintenance of pluripotent stem cells, and inhibition of differentiation [11, 12]. Additionally, FBXO21 has been shown regulate the response to immune stimuli via proinflammatory cytokine mediated pathways through ubiquitination of ASK1 via K29 linkage [10]. *FBXO21* is highly expressed in stem and progenitor (HSPC) population, the tumor initiating population in AML, but has low to no expression in mature myeloid populations [13]. To determine the role of FBXO21 in hematopoietic development we generated a conditional knockout model and crossed it to *Vav1-CRE*, which deletes during early hematopoiesis. Deletion of *Fbxo21* revealed minimal change in the hematopoietic development [13]. However, knockdown (KD) of *FBXO21* in AML patient samples and patient derived cells lines led to apoptosis and decreased proliferation at a greater degree compared to normal human CD34 + HSPC. Silencing of *FBXO21* in AML increased expression of inflammatory cytokines and chemokines, including CXCL10. Further, we utilized a mass spectrometry based proteomic approach to identify p85α as a substrate of FBXO21. p85α is targeted for ubiquitination by FBXO21, and stabilization of p85α leads to apoptosis, differentiation, decreased canonical PI3K signaling, and ERK activation. Taken together, the data suggest that FBXO21 plays a key role leukemia progression and maintenance but has a limited role in normal hematopoiesis suggesting FBXO21 as a potential therapeutic target for drug discovery.

## Results

### Low ubiquitin E3 ligase FBXO21 expression correlates with improved survival in AML

**Fig. 1:**
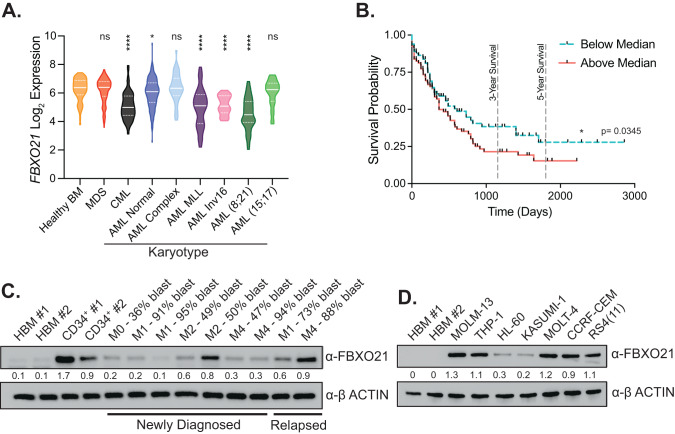
Expression of FBXO21 in AML patients. **A** FBXO21 gene expression analysis of patient samples with different karyotypes from the Leukemia MILE study. **B** Overall patient survival from TCGA AML patient dataset. **C** Western blot of FBXO21 from two independent human BM (HBM) samples, two healthy CD34^+^ samples isolated from mobilized PB, and mononuclear cells from PB of nine AML patients (Supplementary Table 1). **D** Western blot probing for FBXO21 in two independent HBM samples compared to patient-derived AML (MOLM-13, THP-1, HL-60, and KASUMI-1) and ALL (MOLT-4, CCRF-CEM, and RS4(11)) cell lines. (ns non-significant, *p ≤ 0.05, ****p ≤ 0.0001).

Utilizing AML primary patient expression data from the Microarray Innovations in Leukemia study [14], we determined that the expression of the E3 ligase *FBXO21* is significantly downregulated in AML compared to healthy BM (Fig. 1A). The most pronounced decrease in *FBXO21* expression was in patients with mixed lineage leukemia (MLL) and *t*(8;21) translocations (Fig. 1A). Interestingly, lower expression of *FBXO21* is associated with improved survival (Fig. 1B) [6]. Protein expression analysis in mononuclear cells from peripheral blood (PB) of nine AML patients with French-American-British classifications ranging from M0–M4 showed an increase in the levels of FBXO21 compared to two healthy human total BM samples (Fig. 1C). These patients had AML blast counts ranging from 36 to 95%, although only two representative patients relapsed samples had high FBXO21 protein similarly to hematopoietic stem and progenitor population (HPSC) characterized by CD34^+^ expression. In addition, protein expression in patient-derived AML (MOLM-13, THP-1, HL-60, and KASUMI-1) and acute lymphoblastic leukemia (MOLT-4, CCRF-CEM, and RS4(11)) cell lines exhibited high level of FBXO21 compared to two independent human BM samples (Fig. 1D). These findings suggest *FBXO21* expression is associated with prognosis and may contribute to progression leukemia.

### Silencing of FBXO21 in AML alters proliferation, differentiation, and tumor progression

**Fig. 2:**
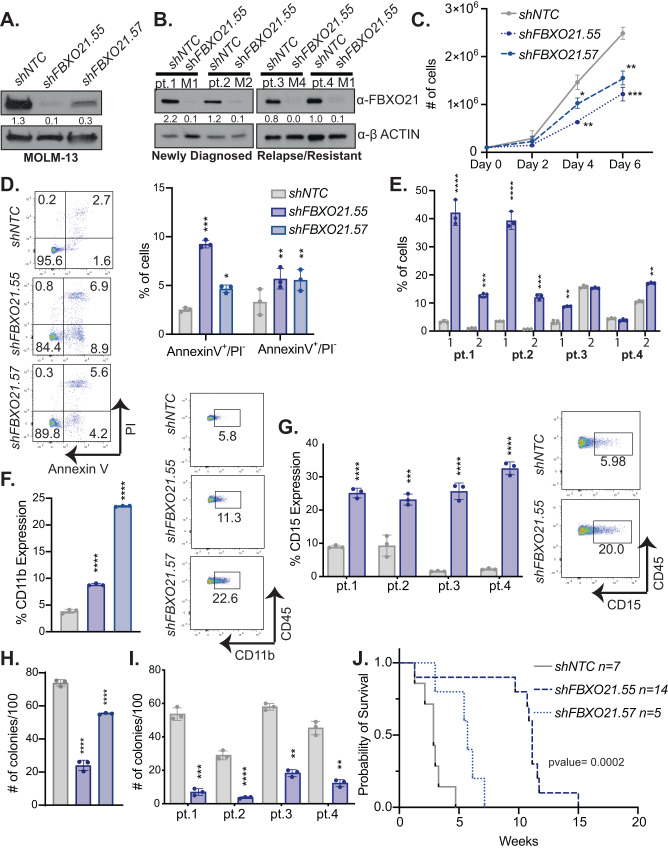
Loss of *FBXO21* alters cell proliferation, differentiation, and survival of AML cells. **A**–**J** (MOLM-13: *n* = 3 biological replicates, AML Patients: *n* = 3 technical replicates) MOLM-13 cells and 4 AML primary samples (2 de novo, 2 relapse) were infected with lentiviral shRNAs against *shFBXO21* and *shNTC* were analyzed at 72 h post puromycin selection by (**A**, **B**) western blot for knockdown in (**A**) AML patient derived cell line, MOLM13, and (**B**) 4 AML primary samples (2 de novo, 2 relapse), (**C**) proliferative ability of MOLM-13 cells by cell count. Cells were stained with (**D**, **E**) (left) representative flowcytometry plot and (right) bar graph of Annexin V and propidium iodide (PI) for percent of (1) AnnexinV^+^/PI^−^ and (2) AnnexinV^+^/PI^+^ apoptotic cells in (**D**) MOLM-13 and (**E**) AML primary cells. Cells were analyzed by flowcytometry for (**F**) (right) representative flowcytometry plot and (left) bar graph of CD11b (MOLM-13) and (**G**) (right) representative flowcytometry plot and (left) bar graph of CD15 (AML primary cells) expression. Colony forming ability by CFU assay in (**H**) MOLM-13 and (**I**) AML primary cells. **J** Survival of sub-lethally irradiated NSG mice transplanted with 5 × 10^5^ MOLM-13 cells infected with shRNAs against *shFBXO21* and *shNTC*. (*p ≤ 0.05, **p ≤ 0.01, ***p ≤ 0.001, ****p ≤ 0.0001).

To understand the role of FBXO21 in AML, we generated *FBXO21* knockdown (KD) AML cells. Here we utilized four patient derived AML cell lines (MOLM-13 (FLT3-IDT/MLL-AF9), THP-1 (MLL-AF9/TP53), Kasumi-1 (AML-ETO/TP53/KIT), and HL-60 (TP53/NRAS)) and 4 independent samples from primary mononuclear PB of AML patients (2 de novo, 2 relapse; Supplementary Table 1), and infected them with lentivirus expressing shRNAs against either a non-targeting control (*shNTC*) or *shFBXO21* to silence *FBXO21* expression (Fig. 2 and Supplementary Fig. 1). Knockdown yielded a 75–93% decrease in FBXO21 protein levels (Fig. 2A, B) and led to a decrease in the cell’s ability to proliferate (Fig. 2C, and Supplementary Fig. 1A, B) and an increase in cells undergoing early (Annexin V^+^/PI^-^) and late (Annexin V^+^/PI^+^) apoptosis (Fig. 2D, E, and Supplementary Fig. 1C). Knocking down *FBXO21* also promoted cell differentiation, indicated by increased expression of CD11b in MOLM-13 cells (Fig. 2F) and CD15 in primary AML cells and AML cell lines, both mature myeloid cell surface markers (Fig. 2G, and Supplementary Fig. 1D). Colony forming potential was decreased following silencing of *FBXO21* when both MOLM-13 cells and primary AML cells were plated in methylcellulose (Fig. 2H, I, and Supplementary Fig. 1E). Importantly, silencing *FBXO21* delayed disease onset of NSG mice following transplantation in comparison to mice transplanted with the *shNTC* in patient derived cell lines, consistent with survival in human AML patients with low *FBXO21* expression (Fig. 2J, and Supplementary Fig. 1F). Combining these findings with previous data showing that patients with low expression of *FBXO21* yields better prognosis suggests that FBXO21 may act as an oncogene in AML.

### Overexpression of FBXO21 leads to increased proliferation and colony formation

**Fig. 3:**
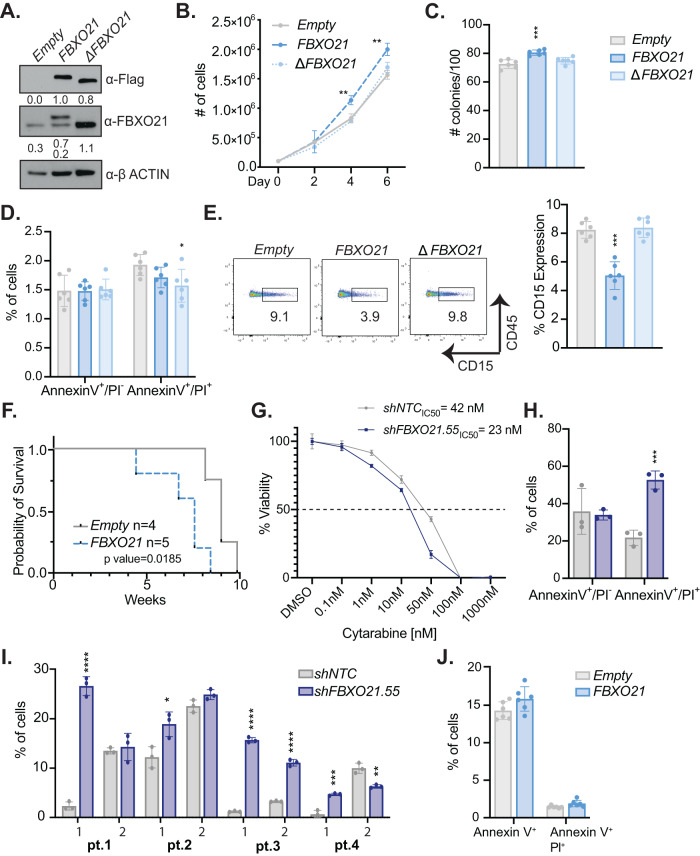
Overexpression of *FBXO21* alters cell proliferation, differentiation, and survival of AML cells. **A**–**F** (*n* = 6, 2 biological, 3 technical replicates) MOLM-13 cells were infected with retrovirus expressing *FBXO21*, *∆FBXO21*, and *Empty* control were analyzed after sorting via FACS for (**A**) protein expression by western blot, (**B**) proliferative ability by cell count, (**C**) colony forming ability by CFU assay, (**D**) for percent of Annexin V^+^/PI^-^ and Annexin V^+^/PI^+^ apoptotic cells, (**E**) (left) representative flowcytometry plot and (right) bar graph of CD15 expression by flow cytometry, and (**F**) survival of sub-lethally irradiated NSG mice transplanted with 5 × 10^5^ cells infected with an *Empty* control or a plasmid overexpressing *FBXO21*. **G** MTT assay in MOLM-13 *NTC* and *shFBXO21.55* following treatment with between 0.1-1000 nM cytarabine for 48 h. **H** MOLM-13, and (**I**) AML primary cells with *FBXO21* KD and *shNTC*, and (**J**) MOLM-13 *FBXO21* and *Empty* were treated with 50 nM cytarabine for 48 h, stained with Annexin V and PI for Annexin V^+^/PI^−^ and Annexin V^+^/PI^+^ apoptotic cells, and analyzed by flow cytometry. (*p ≤ 0.05, **p ≤ 0.01, ***p ≤ 0.001, ****p ≤ 0.0001).

Expression data suggest that higher expression of *FBXO21* is correlated with poor prognosis and relapse. To determine if overexpression leads to increased proliferation, we retrovirally expressed empty vector or flag-tagged FBXO21 to overexpress FBXO21 in tandem with GFP in MOLM-13 cells (Fig. 3A). In addition, we overexpressed FBXO21 with the FBOX domain deleted (*ΔFBXO21*), a catalytically dead mutant of FBXO21 due to FBOX domain required for binding to SKP1. Overexpression of FBXO21 led to increased proliferation and colony formation, whereas ΔFBXO21 overexpression did not alter proliferation or colony formation potential (Fig. 3B, C). Increased expression did not alter apoptosis but led to decreased cell surface expression of CD15 and increased disease onset in NSG mice (Fig. 3D–F). These findings of FBXO21 overexpression displayed an inverse effect from cells having FBXO21 knocked down, where KD had led to increased expression of CD15, decreased colony formation, and a delay in disease onset in NSG mice (Fig. 2G, J). The decrease in survival of NSG mice that received FBXO21 overexpression AML cells correlates with expression data linking patients with higher expression of FBXO21 have poorer survival.

Typically, the first line of treatment for AML is administrating intense induction chemotherapy—generally a combination of cytarabine and an anthracycline such as daunorubicin, with the occasional addition of all trans retinoic acid for certain subtypes [15–18]. To determine whether *FBXO21* expression has an impact on cytarabine sensitivity, we treated *FBXO21* KD or overexpression AML cells with cytarabine. Silencing of FBXO21 led to an additive effect of cytarabine decreasing the IC:50 from 42 nM to 23 nM (Fig. 3G). Following treatment of 50 nM of cytarabine for 48 h, we measured apoptosis of the live cell population by flow cytometry for annexin V. Silencing of *FBXO21* led to increased sensitivity to cytarabine (Fig. 3H, I), whereas cells overexpressing *FBXO21* had no changes in apoptosis and cell death (Fig. 3J). Together these finding demonstrate that levels of FBXO21 impact survival, differentiation, and sensitivity to current therapeutics.

### Silencing of FBXO21 leads to cytokine and chemokine response

**Fig. 4:**
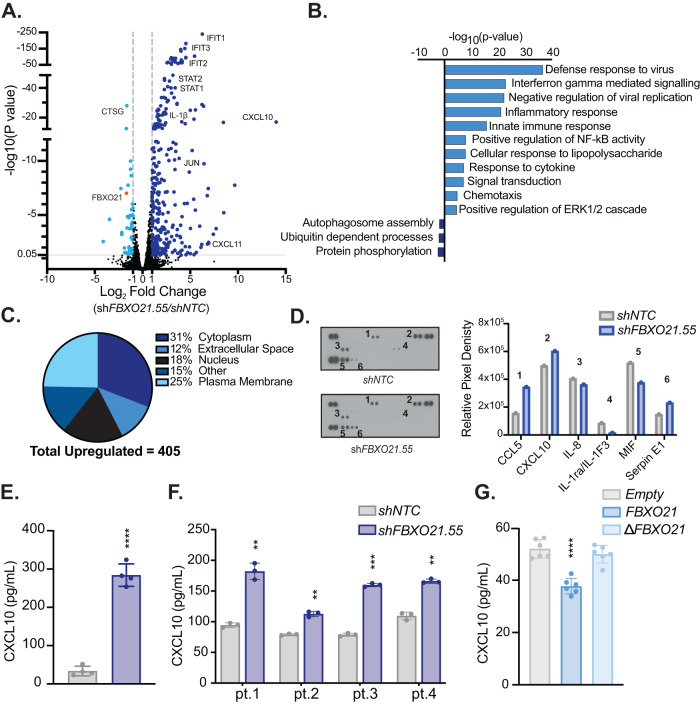
RNASeq data reveals significant increase in cytokine/chemokine levels when *FBXO21* is knocked down. **A** Volcano plot showing fold change of expressed genes from MOLM-13 cells with silenced *shFBXO21* and *shNTC*. **B** Gene ontology analysis showing pathways known to be associated with significantly upregulated (*p* < 0.05, ≥1-fold change) and downregulated (*p* < 0.05, ≤1-fold change) genes using DAVID bioinformatics database. **C** Localization of significantly upregulated genes (405). **D** Cytokine array for supernatant of MOLM-13 cells with *shFBXO21* KD and *shNTC*, membranes showing the change in inflammatory cytokines and quantified intensities relative to internal standards. CXCL10 secretion was evaluated by enzyme-linked immunosorbent assay (ELISA) in (**E**) MOLM-13 cells with silenced *shFBXO21* and *shNTC*, (**F**) AML primary cells with silenced *shFBXO21* and *shNTC*, and (**G**) MOLM-13 cells expressing *FBXO21* and *Empty* control.

To understand transcriptional changes due to depletion of *FBXO21*, we performed RNA-sequencing on MOLM-13 *shNTC* and *shFBXO21* targeted cells. RNA-sequencing data showed that silencing of *FBXO21* led to a dramatic increase in the expression of inflammatory cytokine/chemokine related genes, including *CXCL10*, *CXCL11, IFIT1, IFIT2, IFIT3, IL1β, STAT1, and STAT2* (Fig. 4A). These genes are associated to pathways including inflammatory response, signal transduction, and positive regulation of the ERK1 and ERK2 cascade (Fig. 4B). Differentially expressed RNAs were found in all cellular compartments with ~12% associated with the extracellular space and ~25% associated with the plasma membrane (Fig. 4C). Cytokine arrays confirmed that CCL5 and CXCL10 proteins are also found to be upregulated in the media following silencing of *FBXO21* (Fig. 4D). We identified a 14-fold change increase of *CXCL10* expression at the RNA level and confirmed up-regulation of the protein in both *shFBXO21* KD AML cell lines and primary patient samples (Fig. 4E, F). Interestingly, overexpression of *FBXO21* in MOLM-13 led to a decrease in the amount of CXCL10 in the media (Fig. 4G). These findings suggest that CXCL10 is regulated downstream of FBXO21. Although we have observed an increase in CXCL10 at both RNA and protein levels, we did not observe a change in CXCL10 receptor, CXCR3 at the RNA level (data not shown). CXCL10 is known to be regulated through various MAP kinase pathways, including through the activation of transcription factor NFκB via JNK, p38, ERK1/2, and JAK/STAT [19]. These findings suggest that silencing of *FBXO21* in AML alters cytokine signaling.

### FBXO21 substrate identification in AML

**Fig. 5:**
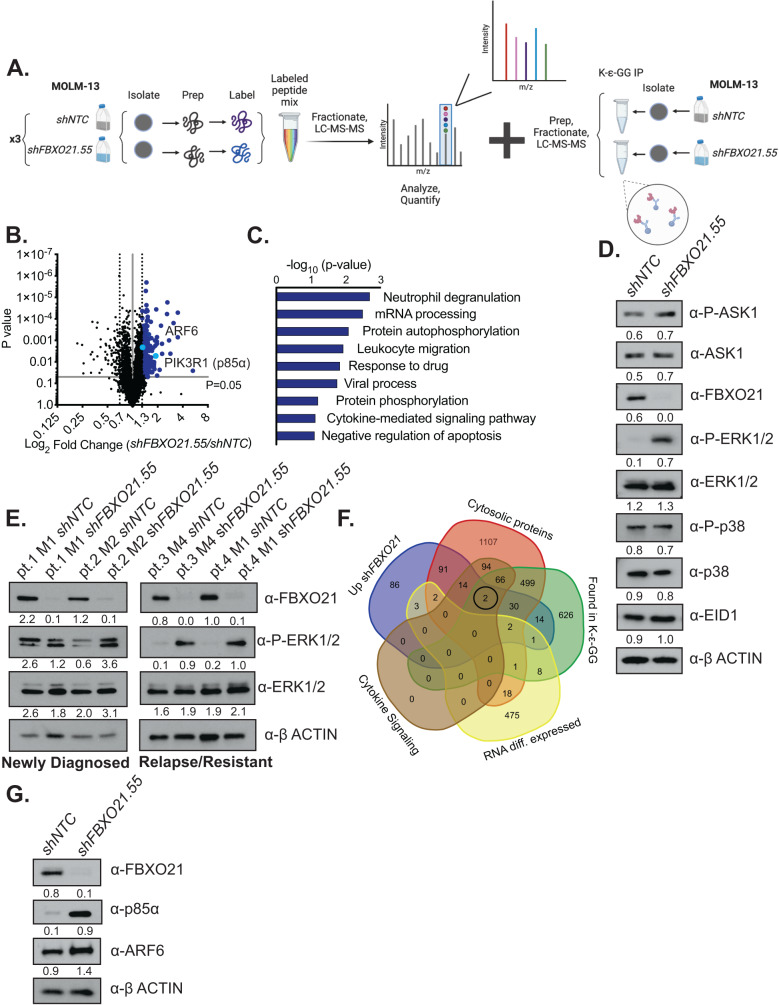
Mass Spectrometry (MS) identifies potential substrate of FBXO21. **A** Schematic of TMT MS and K-ε-GG IP/MS using MOLM-13 cells infected with shRNAs against *shFBXO21* and *shNTC*. **B** Volcano plots showing fold change of expressed proteins from *shFBXO21* compared to *shNTC* cells. **C** Gene ontology analysis showing pathways known to be associated with significantly upregulated (*p* < 0.05, ≥1.3-fold change) proteins using DAVID bioinformatics database. Western blot for previously known FBXO21 substrates (ASK1, EID1) and other MAPK pathway proteins (**D**) in MOLM-13 cells and (**E**) AML primary cells. **F** Venn Diagram of combined TMT MS, K-ε-GG IP/MS, cytosolic proteins, proteins involved in cytokine signaling pathways, and RNA-seq data comparing overlap of differentially expressed proteins and genes. **G** Western blot in MOLM-13 cells for validation of upregulated proteins of interest identified via the combination of proteomic and genomic analysis.

Since FBXO21 is a substrate recognition component of the SKP1-Cullin-FBOX (SCF)-type E3 ligase complex, we performed a combination of mass spectrometry (MS) approaches to identify the substrate of FBXO21 in AML. First, we performed tandem mass tag (TMT) MS, which allowed us to quantitatively identify changes in protein abundance between our MOLM-13 *shFBXO21* KD, and its respective *shNTC* cells (Fig. 5A). In addition, we performed K-ε-GG immunoprecipitation followed by MS on our *shNTC* and *shFBXO21* KD cell lines, which allowed for identification of unique ubiquitination sites through enrichment of Ub-reminant diglycyl-lysine (K-ε-GG) and protein identification by tandem MS (Fig. 5A). Here we identified 260 proteins upregulated following silencing of FBXO21 in TMT MS, including proteins associated with cytokine-mediated signaling pathways and 1297 ubiquitinated peptides in K-ε-GG MS, of which 50 were found either more abundant or only present in the MOLM-13 *shNTC* cell line, which would be predicted for a substrate targeted by polyubiquitination followed for proteasomal degradation (Fig. 5B, C; Supplementary Tables 2, 3).

FBXO21 has only two known substrates in other cell types, EP300-interacting inhibitor of differentiation 1 (EID1) and apoptosis signaling kinase 1 (ASK1, also known as MAP3K5) [7–10]. EID1 has a role in inhibiting differentiation and maintaining the pluripotency of stem cells through interacting with RB1 and EP300 [11, 12]. FBXO21 has also been shown to target ASK1 for K29 ubiquitination in vitro in cell lines regulating response to immune stimuli. However, neither ASK1 nor EID1 were identified in MS approaches, and western blot analysis of *shNTC* and *shFBXO21* KD cells revealed no alterations in EID1, ASK1 activation, or ASK1’s down-stream target, p38; suggesting EID1 and ASK1 are not substrates of FBXO21 in AML (Fig. 5D). In addition, we treated MOLM-13 cells with Selonsertib, a small molecular inhibitor of ASK1. ASK1 inhibition showed that depletion of phosphorylated ASK1 led to diminished p38 phosphorylation, which was not seen following *FBXO21* silencing (Supplementary Fig. 2A). Similar to *shFBXO21* KD, Selonsertib treatment led to decreased proliferation, increased apoptosis, but did not demonstrate the same cell surface phenotype as *shFBXO21* KD further supporting ASK1 is not likely a substrate of FBXO21 in the context of AML (Supplementary Fig. 2B, F).

Interestingly, no changes in total or phospho-p38 was observed, however another MAPK pathway, phospho-ERK1/2 was highly upregulated (Fig. 5D, E). Although, no changes in total ERK1/2 were observed suggesting FBXO21 does not target ERK for poly-ubiquitination, it does not rule out that FBXO21 targets the ERK pathway upstream or an activator of the ERK pathway (Fig. 5D, E). Further supporting that ERK1/2 is not the direct substrate, inhibition of ERK with SCH772984 did not rescue the decrease in colony formation or proliferation in *shFBXO21* KD cells, and CXCL10 levels did not decrease to levels seen in *shNTC* cells (Supplementary Fig. 3). Contrary to previously published worked suggesting ERK activation promotes proliferation, *FBXO21* KD led to decreased proliferation supporting that ERK itself is not a substrate of FBXO21 [20].

Since EID1 and ASK1, the known substrates of FBXO21, showed no alterations in protein abundance, we used a combinational approach to identify potential substrates. Criteria included 1) upregulated at the protein level by TMT MS in *FBXO21* KD cell line (unable to be ubiquitinated and sent to proteasome for degradation), 2) ubiquitin modified as identified in the K-ε-GG mass spectrometry, 3) cytoplasmic proteins since FBXO21 localizes to the cytoplasm, 4) unaltered at the RNA level (as we are identifying modification at the posttranslational level), and 5) proteins associated with cytokine signaling (Fig. 5F, Supplementary Tables 2–5). Crossing the datasets revealed two proteins that meet all five criteria, ARF6 and p85α, both of which were confirmed by western blot to be upregulated following silencing of *FBXO21* in the MOLM-13 cell line (Fig. 5G). In summary, the combination of proteomic and genomic analysis yielded two potential novel FBXO21 substrates in AML.

### FBXO21 targets p85α for ubiquitination and degradation

**Fig. 6:**
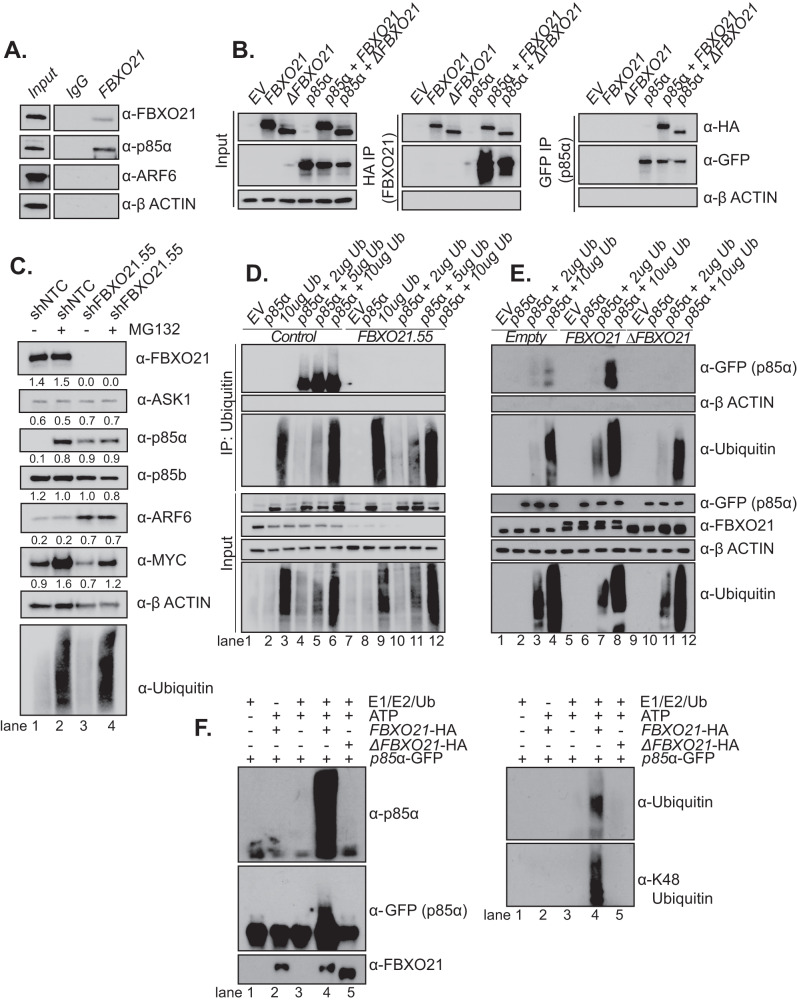
FBXO21 regulates p85α through ubiquitination. **A** Western blot of endogenous immunoprecipitation of FBXO21 in MOLM-13 cell line. **B** Western blot of GFP and HA immunoprecipitation in HEK293T cells transiently transfected with plasmids expressing GFP-tagged *p85α* and/or HA-tagged *FBXO21/∆FBXO21*. **C** Western blot of *shNTC/shFBXO21.55* MOLM-13 cells treated with 20 μM MG132 or DMSO. **D** Western blot of Ubiquitin immunoprecipitation in *shNTC/shFBXO21.55* HEK293T cells transiently transfected with *p85α* and 2, 5, or 10 μg Ubiquitin. **E** Western blot of Ubiquitin immunoprecipitation in *Empty/FBXO21/∆FBXO21* HEK293T cells transiently transfected with *p85α* and 5 or 10 μg Ubiquitin. **F** HEK293T cells were transfected with GFP-tagged *p85α*, HA-tagged *FBXO21*, HA-tagged *ΔFBXO21* as indicated. After immunopurification with anti-GFP/anti-HA, in vitro ubiquitylation of p85*α* was performed in the presence of E1, E2, and ubiquitin (Ub). Samples were analyzed by western blot with the indicated antibodies. *n* = 3 for all experiments.

To determine if either ARF6 and/or p85α are substrates of FBXO21, we performed endogenous IP of FBXO21 in MOLM-13 cells to determine protein interaction. Of the two potential substrates identified through MS approaches, only p85α was found to interact with FBXO21 (Fig. 6A). To further confirm binding of p85α and FBXO21, we transiently expressed p85α tagged with GFP with either wild-type FBXO21 or FBOX domain deleted FBXO21 (ΔFBXO21) tagged with HA in HEK293T, and immunoprecipitated GFP or HA. We found p85α interacted with both full-length and FBOX domain deleted FBXO21 suggesting a direct interaction with FBXO21, and that the interaction is not through the SCF complex (Fig. 6B). To investigate whether p85α is targeted for degradation by the proteasome and whether depletion of FBXO21 blocks degradation, we treated both *shNTC* and *shFBXO21* KD MOLM-13 cells with the proteasome inhibitor MG132. Immunoblotting revealed that in *shNTC* MOLM-13s p85α accumulated in the presence of proteasome inhibitor (lane 1 and 2); however, silencing of *FBXO21* inhibited accumulation of p85α protein following MG132 treatment (lanes 3 and 4) (Fig. 6C). In contrast, ARF6 protein abundance was unaltered in either *shNTC* or *shFBXO21* KD following MG132 suggesting it is likely not regulated in AML by proteasomal degradation and further confirming ARF6 is not a substrate of FBXO21. cMYC was used as a positive control due to known to be targeted by the proteasome in leukemia, and shows accumulation in both *shNTC* or *shFBXO21* KD following MG132 [2].

To further confirm FBXO21 regulates the degradation of p85α, we immunoprecipitated ubiquitin in HEK293T cells stably expressing either *shNTC* or *shFBXO21*, and transiently expressing GFP-tagged p85α, along with increasing concentrations of HA-tagged ubiquitin. Increased ubiquitinated p85α protein was only found in cells expressing FBXO21 (lane 4–6), whereas ubiquitinated p85α was not immunoprecipitated in cells depleted for FBOX21 (lanes 10–12) (Fig. 6D). Similarly, we preformed ubiquitin immunoprecipitation in HEK293T cells stably overexpressing FBXO21 or ΔFBXO21. Overexpression of FBXO21 led to increased immunoprecipitation of ubiquitinated p85α (lane 6–8) compared to endogenous expression of *FBXO21* (lanes 2–4) (Fig. 6E). Deletion of the FBOX domain, due to being catalytically dead and unable to bind the SCF complex, inhibited p85α ubiquitination (lanes 10–12) (Fig. 6E). Finally, we reconstituted the ubiquitination of p85α in vitro. Immunopurified FBXO21, but not FBOX deficient FBXO21 ubiquitinated p85α in vitro (Fig. 6F). Together, these results demonstrate that FBXO21 directly mediates the ubiquitylation and degradation of p85α in AML.

### *p85*α regulates CXCL10 promoting apoptosis and differentiation of AML

**Fig. 7:**
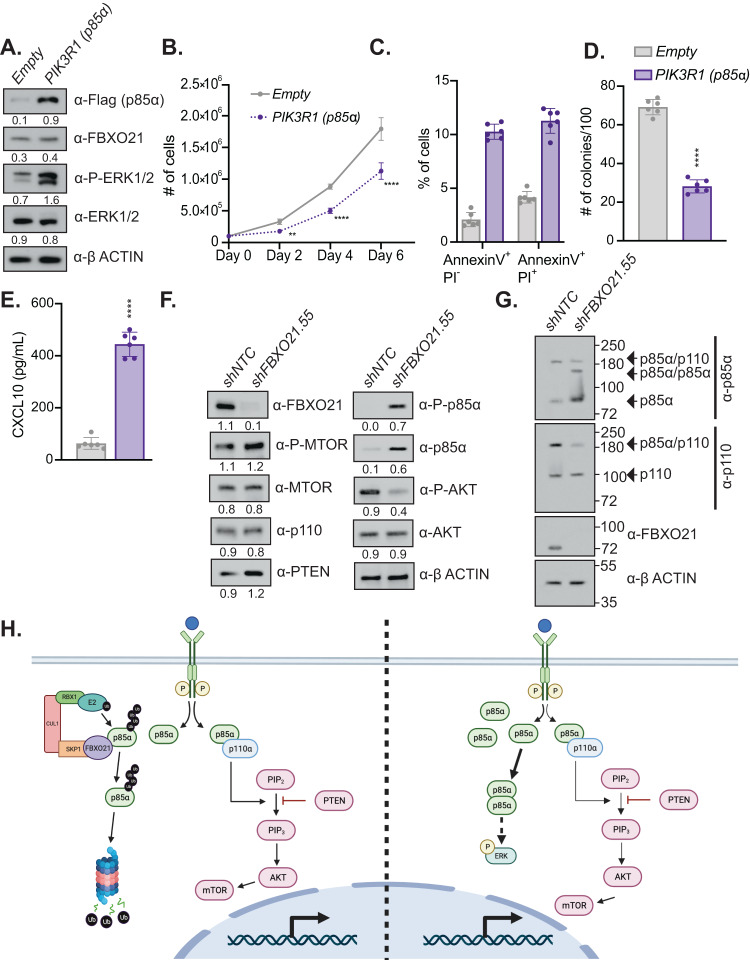
Overexpression of *p85α* mimics FBXO21 KD phenotype in AML cells and leads to altered PI3K pathway activation. (*n* = 6, 2 biological, 3 technical replicates) MOLM-13 cells were infected with retrovirus expressing *p85α* and *Empty* control were analyzed after sorting via FACS by (**A**) western blot, (**B**) proliferative ability by cell count, (**C**) for percent of Annexin V^+^/PI^-^ and Annexin V^+^/PI^+^ apoptotic cells, (**D**) colony forming ability by CFU assay, and (**E**) CXCL10 secretion by ELISA. Western blot in MOLM-13 cells with silenced *shFBXO21* and *shNTC* for (**F**) PI3K pathway proteins and (**G**) native gel for PI3K complex proteins. **H** Schematic highlighting FBXO21 mediated alterations within PI3K signaling pathway.

p85α is part of the PI3K pathway and is a central signaling pathway for hematopoietic cells, and regulates crucial functions such as proliferation, differentiation, and survival [21, 22]. p85α (PIK3R1) and p85β (PIK3R2) are the main regulatory subunits of PI3K which mediate the catalytic activity of p110 [23], however mass spectrometry approaches only found p85α differentially expression and p85β isoform was not found among the proteins expressed (Supplementary Table 2). p85α is significantly upregulated in AML patients and increased expression correlates with a worse survival rate [24]. However, we found high levels of p85α due to silencing of *FBXO21* led to an increase in differentiation, and promoted cell death in AML. This suggests that p85α could work in a dose dependent manner. To determine if increased expression of p85α in MOLM-13 cells contributed to the increased cell death, and decreased proliferation, we stably overexpressed flag-tagged p85α in MOLM-13 cells. Overexpression of p85α increased ERK activation, similarly, to silencing of *FBXO21*, as well as, decreased colony formation, proliferation, and promoted apoptosis (Fig. 7A–D). Corresponding to what was previously shown in our *FBXO21* KD, ERK activation due to p85α overexpression led to elevated CXCL10 (Fig. 7E).

Both silencing of *FBXO21* and overexpression of p85α induced ERK activation and CXCL10 expression. CXCL10 protein expression in *shFBXO21* MOLM-13 cells could be partially rescued by inhibition of ERK suggesting high levels of CXCL10 affected AML survival and proliferation (Supplemental Fig. 3E). Addition of increasing concentration of CXCL10 at dosages corresponding to levels seen with elevated p85α, similarly decreased colony formation and proliferation, and led to increased apoptosis in MOLM-13 cells (Supplemental Fig. 4A–D). Together these finding suggest that p85α overexpression directly leads to ERK activation and elevated CXCL10 expression, and elevated CXCL10 has a negative impact on AML cells.

In canonical PI3K signaling, the catalytic reaction depends on the activity of p110 binding p85α leading to downstream activation of mTOR and AKT [25–28]. However previous studies have revealed free p85 can dimerize, negatively impacting PI3K signaling and activating MAPK pathways [29–31]. *shFBXO21* KD AML cell lines revealed no alterations in mTOR activation following p85α stabilization, however decreased AKT activation was seen with a slightly decreased interaction was seen between p110 and p85α, and dimerization of p85α is found (Fig. 7G). This suggests that loss of FBXO21 leads to decreased canonical PI3K signaling and promotes dimerization of p85α leading to cell death and differentiation of AML cells by elevated CXCL10 via ERK activation.

## Discussion

Collectively, our data suggest a novel role of ubiquitin E3 ligase FBXO21 in mediating AML survival and cytokine signaling pathways via p85α ubiquitylation. Although *FBXO21* RNA is expressed at lower levels of RNA overall, patients with normal or complex karyotypes and MLL translocations had higher expression FBXO21 protein than healthy BM. Higher expression of FBXO21 correlates with poor survival, which fits with patients with both complex karyotypes and MLL translocations being associated with poor survival [32–34]. Silencing in both primary patient samples and AML derived cell lines revealed FBXO21 is required for proliferation, and survival of AML cells. We utilized 4 different AML cell lines, including MOLM-13, which contains a MLL translocation and by protein showed the highest expression of FBXO21. Although MOLM-13 showed significant alterations in vivo, the other cell lines similarly demonstrated that loss of FBXO21 led to decreased proliferation, and increased apoptosis/cell death suggesting FBXO21 could not only be a target in patients with high expression of FBXO21 but all patients. We also showed previously that alterations to FBXO21 minimally affects steady-state hematopoiesis. Silencing of FBXO21 in primary human CD34+ cells showed only ~50% reduction in colony-formation with no induction of early apoptosis, whereas in primary AML cells there was ~90% reduction in colony formation and up to 40% induction of early apoptosis suggesting a therapeutic window for targeting FBXO21 in the context of AML. These findings suggest targeting FBXO21 could solely affect the AML tumor cells with minimal toxicity to the remaining healthy hematopoietic cells [13].

Tight regulation of cytokine signaling within the BM is vital for normal hematopoiesis, and disruptions in cytokine signaling exert profound effects on disease progression and survival of AML. Cytokine and chemokine production in AML is known to be induced by the activation of MAP kinase pathways, and through silencing of *FBXO21* in AML, we have observed modulation of the MAP kinase pathway, ERK1/2. The ERK pathway has been previously found to play a major role in the differentiation and proliferation of myeloid cells by regulating inflammatory cytokines and chemokines [35, 36]. Total ERK protein expression was unchanged following silencing of *FBXO21* suggesting ERK itself is not the substrate of FBXO21. However mass spectrometry combined with our genomic dataset revealed p85α (*PIK3R1)* as the ubiquitination target of *FBXO21*. p85α (*PIK3R1)* expression is significantly upregulated in AML and increased expression correlates with a worse survival rate, and PI3K pathway is among one of the most frequently upregulated intracellular pathways [6, 37, 38]. Our studies revealed overexpression of p85α was detrimental to the AML cells suggesting that too much p85α also impacts the survival and proliferation of AML. In previous studies we demonstrated that in chronic myeloid leukemia (CML) the oncogene cMYC was regulated by FBXW7 and in a similar fashion silencing of FBXW7 led to stabilization of cMYC which was toxic to the CML cells suggesting specific dosage of oncogenes may be required to drive the disease [2].

Although p85α does not directly bind ERK, it has been suggested that there is crosstalk between the two pathways and that they are not linear signaling cascades (Fig. 7H) [39–41]. Overexpression of p85α in MOLM-13 cells, similar to silencing of *FBXO21*, led to activation of ERK and increased CXCL10 suggesting that p85α is either indirectly or directly activating ERK. In addition, we found that silencing of *FBXO21* leads to decreased canonical PI3K signaling with decreased activation of AKT, as well as decreased interaction with the catalytic sub-unit p110. Excess free p85α due to overexpression and stabilization of p85α protein promoted dimerization of p85α. Future studies are needed to decipher the signaling cascade altered by FBXO21 silencing and p85α overexpression. It is unknown whether p85α dimers can activate ERK or decreased AKT phosphorylation promotes ERK activation. Addition of CXCL10 to the media led to an anti-proliferative effect, however previous studies have shown inhibition of AKT signaling can also inhibit suggesting multiple players in the signaling cascade maybe contributing to the anti-proliferative effects. These findings identify a novel role of FBXO21 in regulating PI3K signaling in AML.

These findings identify a novel role for FBXO21 in PI3K signaling by ubiquitination of p85α. Although p85α is a regulatory sub-unit of PI3K signaling and lacks kinase activity, excess free p85α following silencing leads to dimerization and decreased interaction with its catalytic sub-unit p110. Taken together, the data suggest targeting FBXO21 could inhibit canonical PI3K signaling and impedes growth of AML, but may also impede growth of other cancer sub-types dependent on canonical PI3K signaling.

## Materials and methods

### Cell culture/transplantation

HEK293T, PHOENIX-Amphos, HL-60, KASUMI-1, THP-1, and MOLM-13 cells were purchased from ATCC and DSMZ. Human AML cells (Cureline Translational Cro) were cultured in StemSpan SFEM II media with CD34^+^ Expansion Supplement (StemCell Technologies). The shRNA plasmids were purchased from Sigma-Aldrich. *FBXO21* and *∆FBXO21* were subcloned from pCDNA (kind gift from Dr. Yukiko Yoshida, Tokyo Metropolitan Institute of Medical Science) into pMIGR1 with tandem Strep and Flag tags, and retrovirus was produced as previously described [8, 42]. Lentivirus was produced according to manufacturer instructions. Cells were treated with 1 μg/ml of puromycin 48 h post infection. For cytarabine treatment, cells were treated for 48 h with 50 nM cytarabine, and for MG132 treatment, cells were treated for 4 h with 20 µM MG132. For CFC assay cells were plated 100 cells/well of a 24-well plate in Methocult (H4434, StemCellTechnologies, Vancouver, BC, Canada), and counted between day 7-10.

For transplants, 5 × 10^5^ MOLM-13 cells were transplanted into sub-lethally (250 cGy) irradiated 6–10 week old NSG mice (#005557, Jackson Labs). All experiments performed were approved by the Institutional Animal Care and Use Committee of the University of Nebraska Medical Center in accordance with NIH guidelines.

### Flow cytometry analysis

For apoptosis, staining was performed following BioLegend apoptosis staining protocol. Antibodies listed in supplemental materials and methods.

### RNA-sequencing

Total RNA was harvested from cells using the Monarch Total RNA Miniprep Kit (New England Biolabs, Ipswich, MA, USA). RNA sequencing and analysis was performed by Novogene.

### Cytokine and ELISA assays

Cytokine arrays were performed in accordance with the manufacturer’s protocol (R&D Systems, Proteome Profiler Array: Human Cytokine Array Kit). CXCL10 ELISA assays were performed per manufacturer’s protocol (R&D Systems, Human CXCL10/IP-10 DuoSet ELISA).

### TMT labeling and mass spectrometry

Samples were prepared and TMT-labeled per manufacturer’s protocol (ThermoFisher TMT16plex Mass Tag Labeling Kits). Following TMT labeling, acetonitrile was removed by speedvac, and samples were resuspended in 0.1% trifluoroacetic acid. Sample concentrations were re-quantified (Pierce Quantitative Colorimetric Peptide Assay kit) and combined in equal concentration. Following combination, samples were fractionated by ThermoFisher high pH reverse phase fractionation kit following manufacturer’s protocol. Fractions were dried and resuspended in 0.1% Formic Acid for MS analysis as previously described [43, 44]. K-ε-GG immunoprecipitation followed by mass spectrometry was performed according to manufacturer’s protocol (Cell Signaling, PTMScan® Ubiquitin Remnant Motif (K-ε-GG) Kit #5562) [45].

### In vitro ubiquitination

HEK293T cells were transfected with plasmids encoding HA-*FBXO21*, HA-*ΔFBXO21*, or GFP-*p85α*. 48 h post transfection, HA-*FBXO21*, HA-*ΔFBXO21*, or GFP-*p85α* were immunopurified from the whole cell extracts using Anti-HA (Sigma) or Anti-GFP (MBL International) beads overnight at 4 °C. The immunopurified HA-*FBXO21* or HA-*ΔFBXO21* (0.5 µg) proteins were incubated with immunopurified GFP-*p85α* (0.5 µg), E1 (500 ng), E2-UbcH5a (500 ng), FLAG-ubiquitin (0.5 µg) (BostonBiochem), and ATP (10 mM). Ubiquitylation reactions were performed in 100 mM NaCl, 1 mM DTT, 5 mM MgCl_2_, 25 mM Tris-Cl (pH 7.5), incubated at 30 °C for 2 h, and stopped with 2× laemmli buffer (10 min at 95 °C).

### Statistical analysis

All experiments were performed in triplicate unless noted and statistical analyses were performed using unpaired two-tailed Student’s *t* test assuming samples of equal variance. Error bars depict the standard deviation ± mean. For survival curve, the *p* value was calculated using a Log-rank (Mantel-cox) test.

### Supplementary information

Suuplemental material

Supplemental Tables

## Data Availability

The datasets generated during and/or analyzed during the current study are available in the ProteomeXchange repository, PXD04419246 [46]. The remaining data needed to evaluate all conclusions are available within the Article and/or Supplementary Information.
