# Supplementary material for: FBXO21 mediated degradation of p85α regulates proliferation and survival of acute myeloid leukemia
Source: Leukemia. 2023 Sep 9;37(11):2197–208. doi: 10.1038/s41375-023-02020-w (PMC10624613; doi:10.1038/s41375-023-02020-w)
Supplement: Supplementary file 1 — Suuplemental material [file 41375_2023_2020_MOESM1_ESM.pdf]

Weber, Wittorf et al. Supplemental Figure 1

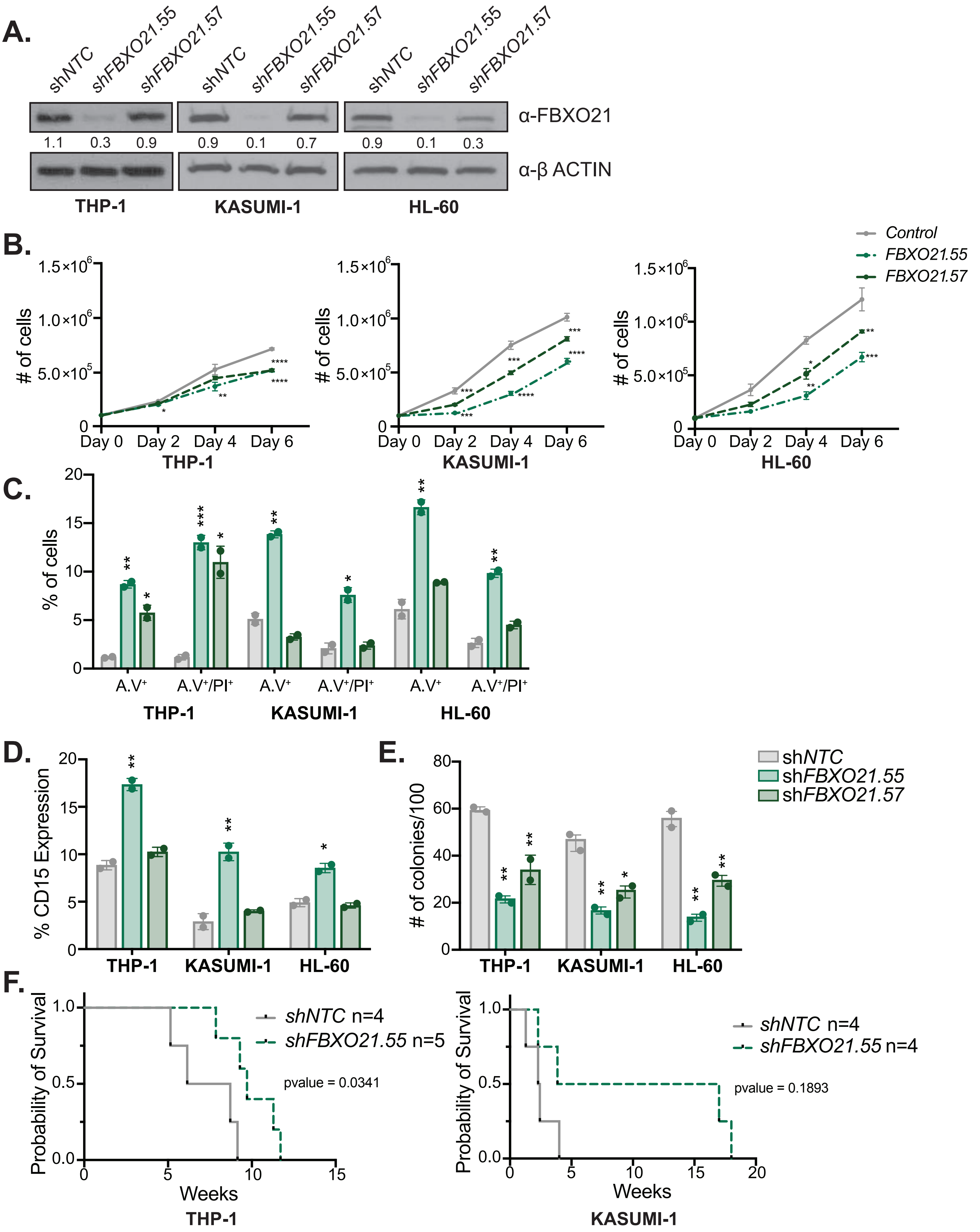

Weber, Wittorf et al. Supplemental Figure 2

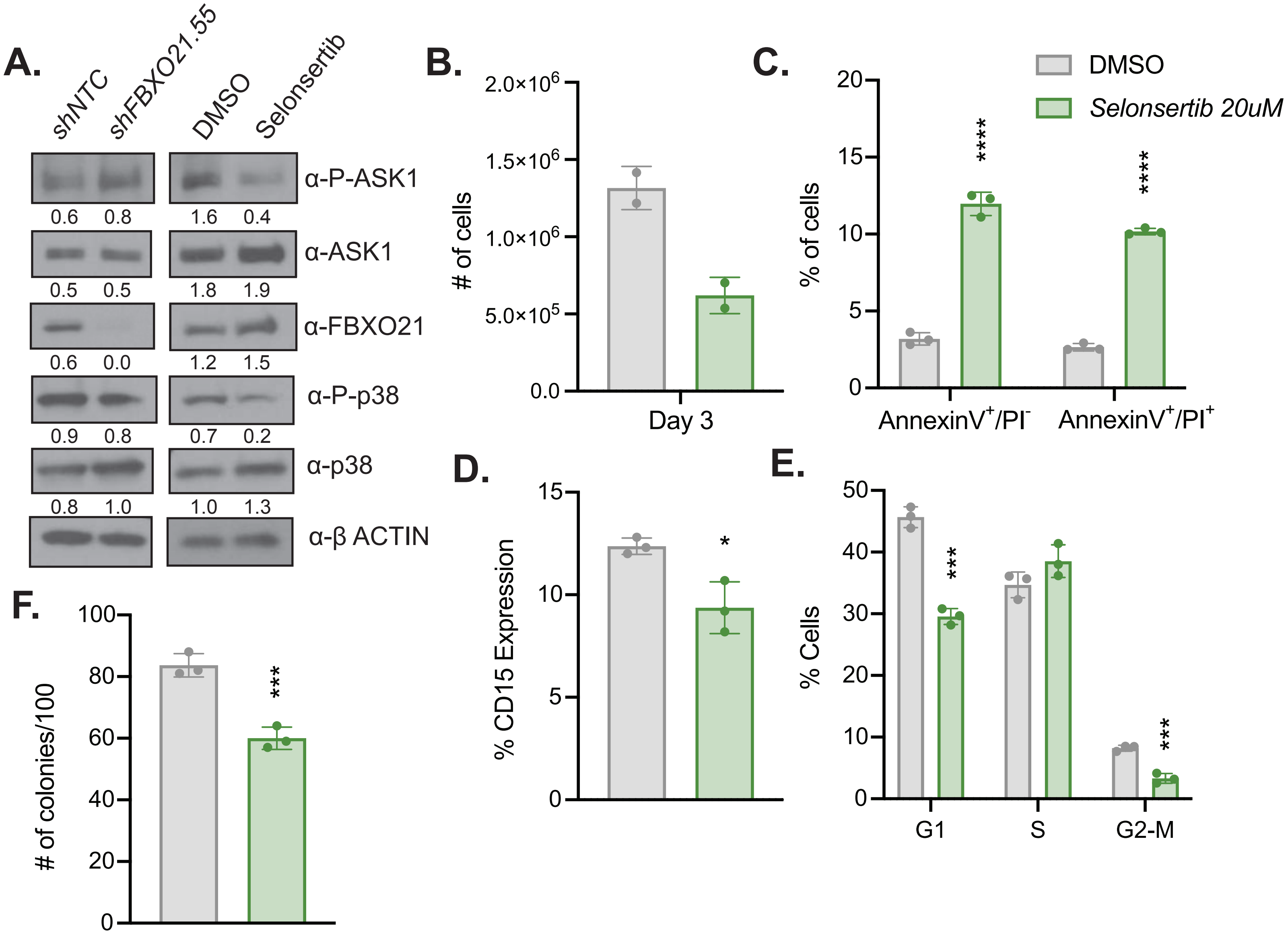

Weber, Wittorf et al. Supplemental Figure 3

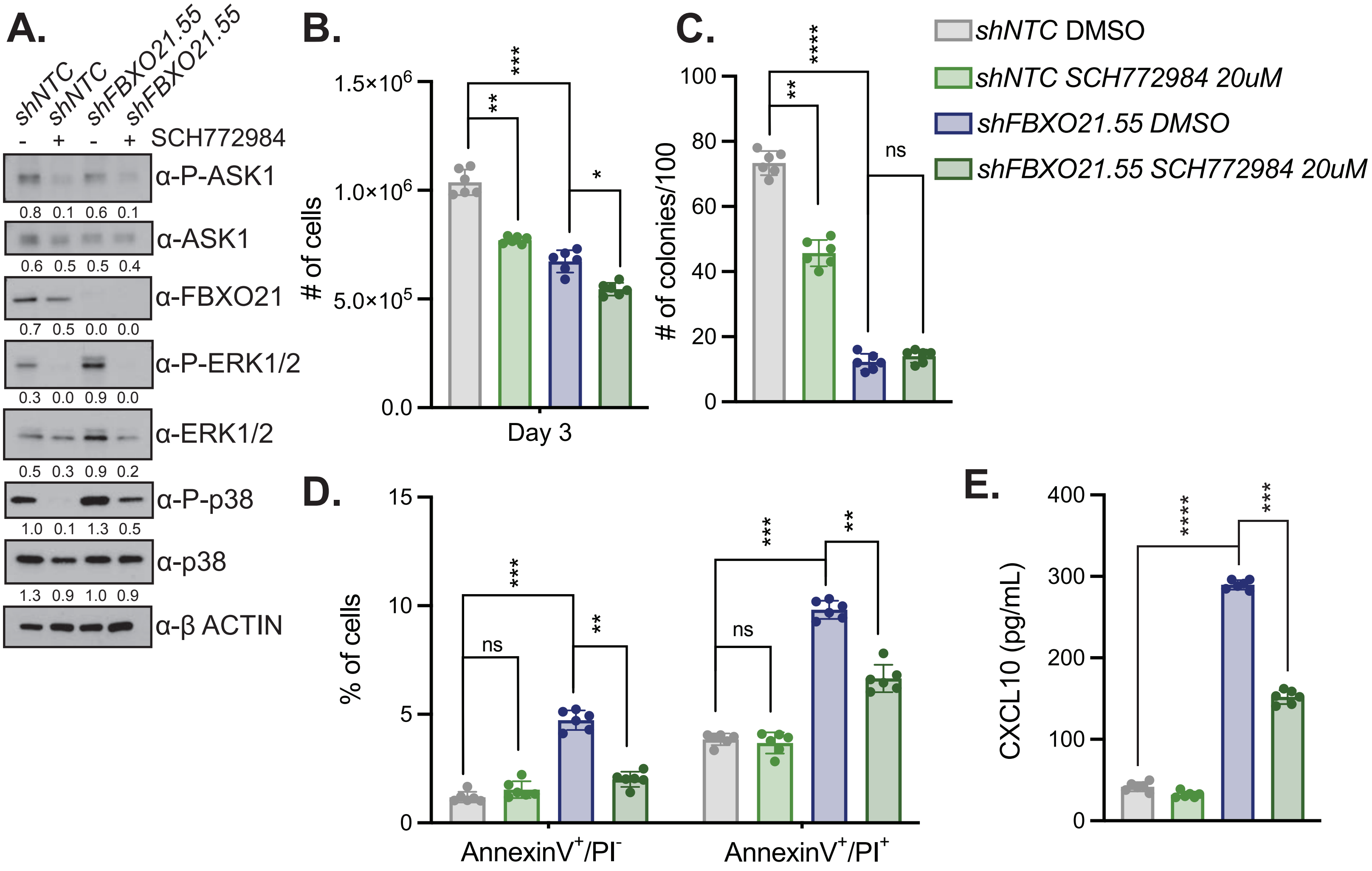

Weber, Wittorf et al. Supplemental Figure 4

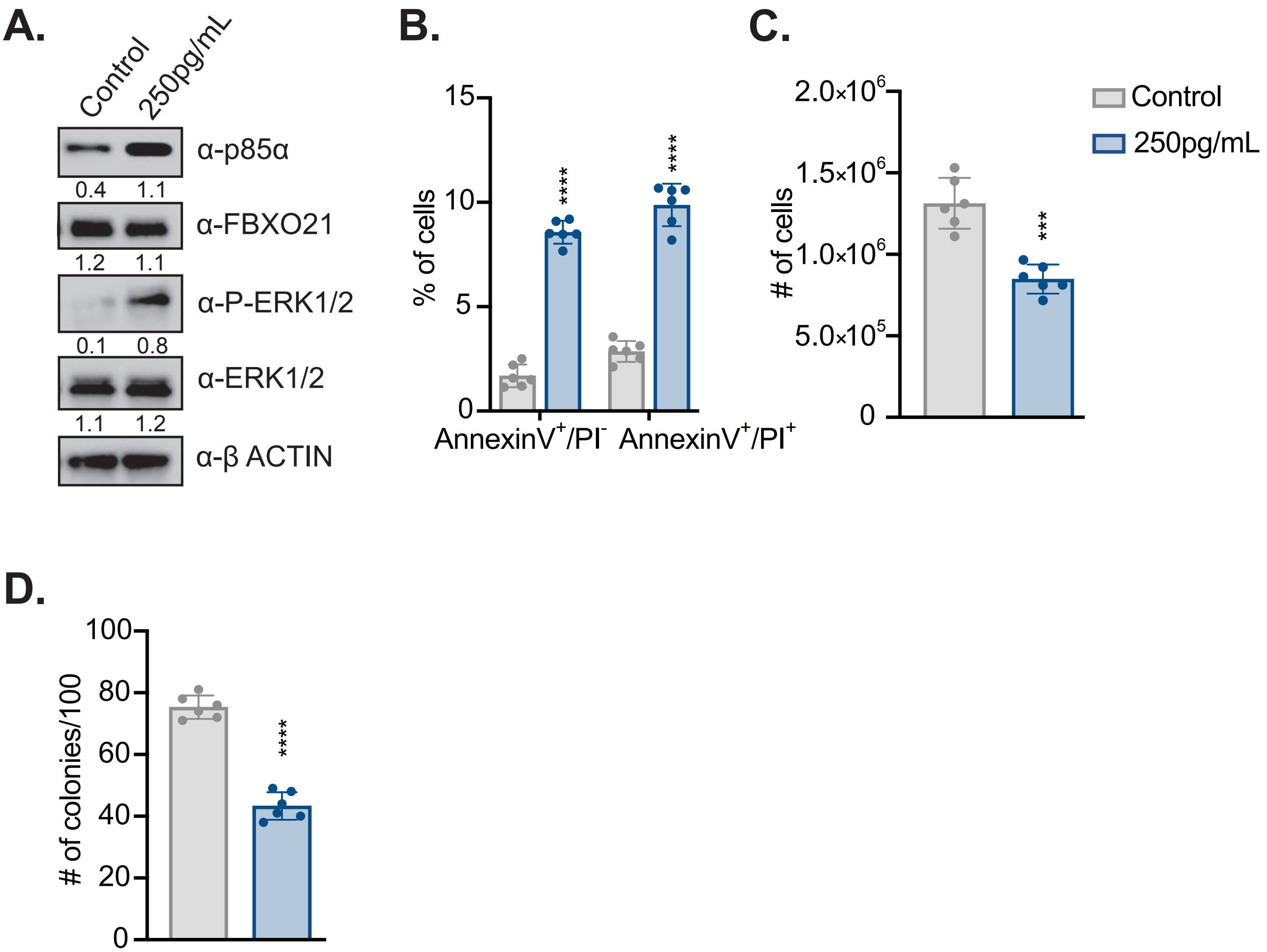

SUPPLEMENTAL MATERIALS AND METHODS

Supplementary Figure 1. Loss of FBXO21 in AML cell lines alters growth, differentiation, and survival. A-F (n=2 biological replicates) THP-1, Kasumi-1 and HL-60 were infected with lentiviral shRNAs against *FBXO21* and non-targeting control (*shNTC*) were analyzed at 72 hours post puromycin selection by **A** western blot for knockdown **B** proliferative ability cells by cell count. **C-D** Cells were also analyzed by flow cytometry with **D** Annexin V and propidium iodide (PI) for percent of Annexin V^+/PI^- and Annexin V^+/PI^+ apoptotic cells and **E** CD15 expression. **E** Cells were plated in CFU assay to analyze colony forming ability. **F** Survival of sub-lethally irradiated NSG mice transplanted with $5x10^5$ cells infected with shRNAs against *FBXO21* and *shNTC*. (* $p \leq 0.05$, ** $p \leq 0.01$)

Supplementary Figure 2. Inhibition of ASK1 activation does not lead to differentiation in AML cells. A-F (n=3 biological replicates) MOLM-13 cells were treated with 20 µM Selonsertib for 72 hours and analyzed for **A** protein abundance by western blot in comparison to *shNTC/shFBXO21.55* cells, **B** proliferative ability by cell count after 72 hours (n=2 biological replicates), and **C** Annexin V and propidium iodide (PI) by flow cytometry for percent of Annexin V^+/PI^- and Annexin V^+/PI^+ apoptotic cells. **D-E** Cells were also analyzed by flow cytometry for **D** CD15 expression and **E** cell cycle progression via DAPI/Ki67 staining. **F** Cells were analyzed for colony forming ability by CFU assay. (* $p \leq 0.05$, ** $p \leq 0.01$, *** $p \leq 0.001$, **** $p \leq 0.0001$)

Supplementary Figure 3. ERK1/2 inhibition does not rescue *FBXO21* knockdown phenotype. A-F (n=6, 2 biological replicates, 3 technical replicates) MOLM-13 cells with *shNTC* or *shFBXO21.55* were treated with 20 µM SCH772984 for 72 hours and analyzed for **A** protein levels by western blot, **B** proliferative ability by cell count after 72 hours, and **C** colony forming ability by CFU assay. **D** Cells were also analyzed by flow cytometry Annexin V and propidium iodide (PI) for percent of Annexin V^+/PI^- and Annexin V^+/PI^+ apoptotic cells. **E** Cell supernatant after 72 hour treatment was analyzed for CXCL10 levels via ELISA. (* $p \leq 0.05$, ** $p \leq 0.01$, *** $p \leq 0.001$, **** $p \leq 0.0001$)

Supplementary Figure 4. CXCL10 stimulation mimics FBXO21 KD phenotype in AML cells. A-D (n=6, 2 biological, 3 technical replicates) MOLM-13 cells were stimulated with 0 (H_2O equal volume control) or 250pg/mL recombinant CXCL10 for 48 hours after 4 hours of serum starvation and analyzed by **A** western blot, **B** for percent of Annexin V^+/PI^- and Annexin V^+/PI^+ apoptotic cells, **C** proliferative ability by cell count after 48 hours of CXCL10 treatment, and **D** colony forming ability by CFU assay. (* $p \leq 0.05$, ** $p \leq 0.01$, *** $p \leq 0.001$, **** $p \leq 0.0001$)

Supplementary Table 1. AML patient data characteristics.

Sample	Sex	Age at Diagnosis	Stage FAB	Peripheral Blood Blast Count	Diagnosis	Karyotype
1	F	35	M0	36%	De novo	Unknown
2	F	66	M1	91%	De novo	Unknown
3	F	66	M1	95%	De novo	Unknown
4	F	54	M2	49%	De novo	Unknown
5	F	51	M2	50%	De novo	46,XX
6	F	73	M4	47%	De novo	Unknown
7	M	65	M4	94%	De novo	Unknown
8	M	54	M1	73%	Relapse/Therapy Resistant	46, XY
9	M	23	M4	88%	Relapse/Therapy Resistant	t(6,9) (p23,q34)

shRNA	Sequence
pLKO.1 Non-Target Control	5’ – CAA CAA GAT GAA GAG CAC CAA– 3’
sh*FBXO21*. 299755	5’ – CCT GGA CAT CTT TGA CTA CAT – 3’
sh*FBXO21*. 299757	5’ – CGG GCT CAT TAT GAA GCA TAA – 3’

Western Blot Antibodies:

Antibody	Company	Ref#	Dilution
AKT	Cell Signaling	9272	1:1000
Phospho-AKT	Cell Signaling	4060	1:1000
ASK1	Abcam, Cell Signaling	[EP553Y] (ab45178), 8662	1:1000
Phosho-ASK1	Cell Signaling	3764	1:1000
ARF6	Cell Signaling	5740	1:1000
β-actin-HRP	Santa Cruz	sc-47778	1:10000
EID1	Proteintech	11734-1-AP	1:1000
ERK 1/2 (p44/42 MAPK)	Cell Signaling	4695	1:5000
Phospho-ERK [p-p44/42 MAPK] (T202/Y204)	Cell Signaling	4370	1:5000
FBXO21	Abcam	[EPR13163] (ab179818)	1:5000
FLAG-HRP	Sigma	A8592	1:10000
GFP	Cell Signaling	2555	1:5000
HA-HRP	Cell Signaling	14031	1:10000
mTOR	Cell Signaling	2983	1:5000

Phospho-mTOR (Ser2448)	Cell Signaling	5536	1:5000
c-Myc/n-Myc	Cell Signaling	13987	1:1000
p38 MAPK	Cell Signaling	8690	1:5000
Phospho-p38 MAPK (T180/Y182)	Cell Signaling	4511	1:5000
PI3 Kinase p85	Cell Signaling	4257	1:1000
PI3 Kinase p85b	Abcam	[EPR18416] (ab180967)	1:1000
Phospho-PI3 Kinase p85	Cell Signaling	4228	1:1000
PI3 Kinase p110α	Cell Signaling	4255	1:1000
PTEN	Cell Signaling	9552	1:1000
Ubiquitin-HRP	Cell Signaling	14049	1:10000
K48 Ubiquitin	Cell Signaling	8081	1:10000

Flow Cytometry Antibodies

Antibody	Fluorochrome	Clone	Company
Annexin V	APC	n/a	BioLegend
CD11b	APC, BV510	M1/70	BioLegend
CD15	Alexa 647, BV421	MC-480	BioLegend
Ki67	FITC	16A8, 11F6	BioLegend

Class	MILE Study Diagnosis	No of Samples
HBM	Non-leukemia and healthy bone marrow	74
MDS	Myelodysplastic syndrome	206
Normal	AML with normal karyotype	351
Complex	AML with complex karyotype	48
inv(16)	AML with inv(16)/t(16;16)	28
t(15;17)	AML with t(15;17)	37
t(8;21)	AML with t(8;21)	40
MLL	AML with t(11q23)/MLL	38
CML	Chronic myeloid leukemia	76
Total		898

FAB Subtype	TCGA Study Diagnosis	No of Samples
AML M0	AML with minimal maturation	15
AML M1	AML without maturation	41
AML M2	AML with maturation	42
AML M3	Acute promyelocytic leukemia	15
AML M4	Acute myelomonocytic leukemia	37
AML M5	Acute monoblastic or monocytic leukemia	22
AML M6	Acute erythroid leukemia	2
AML M7	Acute megakaryoblastic leukemia	3
Other	Other subtype	2
Total		179

Patient datasets and Human Primary AML Cells

FBXO21 expression and survival analyses in patients utilized data from the publicly available Microarray Innovations in Leukemia (MILE)[33] and TGCA Genomic and Epigenomic Landscapes of Adult De Novo Acute Myeloid Leukemia[34] studies.

Viral transduction and additional cell culture

Lentivirus was made through Lipofectamine 2000 transfection of HEK293T cells, with shRNA plasmid, Pax2, and pMD2.g plasmids, and retrovirus was made through Lipofectamine 2000 transfection of Phoenix-AMPHO cells with pMIG plasmid. Transfection media was changed after 24 hours, and viral media was collected and concentrated after 48 hours. AML cell lines and patient samples were transduced with virus for 1 hour at 1000xg and 37°C. 1-4 million cells were resuspended in 500μL media in a 15mL conical tube with 100µL concentrated virus and 1µL polybrene (10mg/mL). Cells were then resuspended in viral media, plated in 24 well plate, and left to rest for 3 hours at 37°C. After 3 hour rest, cells were resuspended in media and treated with 1µg/ml of puromycin 48 hours post infection or sorted using fluorescent selection marker. AML primary cells were thawed and cultured overnight prior to viral transduction. AML primary cells were cultured in the following media: StemSpan SFEM II, CD34+ Expansion Supplement, and UM729 (1µM).

Western blot analysis and immunoprecipitation

For western blot analysis, samples were lysed in lysis buffer (20mM Tris pH7.5, 150mM NaCl, 1mM EDTA) containing 1X Halt Protease and Phosphatase Inhibitor Cocktail (ThermoFisher, Waltham, MA, USA) and 10 mM N-ethylmaleimide. For native gel western blot analysis, cells were lysed as described above and loaded with 2x sample buffer (62.5mM Tris-HCl, pH 6.8, 40% glycerol, 1% bromophenol blue) and Tris/Glycine running buffer (25mM Tris, 192mM glycine, pH 8.3). Antibodies are listed in supplemental materials and methods. Quantification is reflective of relative fold change as a ratio of each protein band relative to our loading control, β-actin utilizing relative densitometry calculated with ImageJ Software.

For endogenous interaction, protein extracts were incubated with FBXO21 antibody or rabbit IgG control (Cell Signaling Technologies, Danvers, MA) overnight at 4°C, followed by incubation with Protein A agarose beads (Cell Signaling, #9863) for 3 hours at 4 °C. For transient HEK293T transfection, cells were transfected and, cultured for 48 hours prior to lysis. Lysates were incubated with Anti-HA (Sigma #A2095) or Anti-GFP (MBL International #D153-11) beads as described. Beads were washed, and eluted by boiling in 1x laemmli buffer for 5 min.

ASK1 and ERK1/2 inhibitor treatment

MOLM-13 cells were treated for 72 hours with 20 µM Selonsertib (GS-4997, Selleck Chem, S8292) or SCH772984 (Selleck Chem, S710) or equal volume of DMSO (solvent) control.
